# Diagnostic value of multiple diagnostic methods for lymph node metastases of papillary thyroid carcinoma: A systematic review and meta-analysis

**DOI:** 10.3389/fonc.2022.990603

**Published:** 2022-11-10

**Authors:** Si-Rui Wang, Qiao-Li Li, Feng Tian, Jun Li, Wen-Xiao Li, Ming Chen, Tian Sang, Chun-Li Cao, Li-Nan Shi

**Affiliations:** ^1^ Department of Ultrasound, the First Affiliated Hospital of Medical College, Shihezi University, Shihezi, Xinjiang, China; ^2^ NHC Key Laboratory of Prevention and Treatment of Central Asia High Incidence Diseases (First Affiliated Hospital, School of Medicine, Shihezi University), Shihezi, Xinjiang, China; ^3^ Department of Neurology, the First Affiliated Hospital of Medical College, Shihezi University, Shihezi, Xinjiang, China

**Keywords:** lymph nodes metastasis, diagnostic value, network meta-analysis, multiple diagnostic methods, papillary thyroid carcinoma

## Abstract

**Objective:**

This study compared the diagnostic value of various diagnostic methods for lymph node metastasis (LNM) of papillary thyroid carcinoma (PTC) through network meta-analysis.

**Methods:**

In this experiment, databases such as CNKI, Wanfang, PubMed, and Web of Science were retrieved according to the Cochrane database, Prisma, and NMAP command manual. A meta-analysis was performed using STATA 15.0, and the value of the surface under the cumulative ranking curve (SUCRA) was used to determine the most effective diagnostic method. Quality assessments were performed using the Cochrane Collaboration’s risk of bias tool, and publication bias was assessed using Deeks’ funnel plot.

**Results:**

A total of 38 articles with a total of 6285 patients were included. A total of 12 diagnostic methods were used to study patients with LNM of PTC. The results showed that 12 studies were direct comparisons and 8 studies were indirect comparisons. According to the comprehensive analysis of the area of SUCRA, US+CT(86.8) had the highest sensitivity, FNAC had the highest specificity (92.4) and true positive predictive value (89.4), and FNAC+FNA-Tg had higher negative predictive value (99.4) and accuracy (86.8). In the non-invasive method, US+CT had the highest sensitivity, and the sensitivity (SEN) was [OR=0.59, 95% confidence interval (CI): (0.30, 0.89]. Among the invasive methods, the combined application of FNAC+FNA-Tg had higher diagnostic performance. The sensitivity was [OR=0.62, 95% CI: (0.26, 0.98)], the specificity (SPE) was [OR=1.12, 95% CI: (0.59, 1.64)], the positive predictive value was [OR=0.98, 95% CI: (0.59, 1.37)], the negative predictive value was [OR=0.64, 95% CI (0.38, 0.90)], and the accuracy was [OR=0.71, 95% CI: (0.31, 1.12)].

**Conclusion:**

In the non-invasive method, the combined application of US+CT had good diagnostic performance, and in the invasive method, the combined application of FNAC+FNA-Tg had high diagnostic performance, and the above two methods were recommended.

## 1 Introduction

Thyroid cancer (TC) is one of the most common endocrine tumors worldwide, with an incidence rate of 3.1% and a mortality rate of 0.4% (1). The increase in papillary thyroid carcinoma (PTC) is the main reason for the increased incidence of adenocarcinoma (2). Although PTC has a good prognosis, the probability of distant cervical lymph node metastasis (LNM) in PTC patients reaches 90% (3), so the status of LN is also an important basis for judging recurrence and LN dissection (4). At the same time, for patients with LNM of PTC, the operation caused by persistent LNs recurrence will increase the risk of postoperative complications such as dyspnea, asphyxia, hypoparathyroidism, etc. (5, 6). Therefore, preoperative diagnosis is an important means for PTC patients to avoid persistent LNs recurrence and reduce complications.

At present, the commonly used methods for diagnosing the metastasis of LNM of PTC include CT, magnetic resonance imaging (MRI), IWBS, US, strain elastography (SE-US), Contrast-Enhanced Ultrasonography (CEUS), FNAC and other detection methods. But each of these methods has its advantages and disadvantages; In a single diagnostic method, ultrasound (US) is the main basis for clinical diagnosis of PTC (7), and the accuracy of the ultrasound results in the neck is high (8). However, the identification of LNM of PTC often needs to be judged by doctors’ experience, which is often regarded as an inaccurate method (9). For patients with lymph node infiltration or distant metastasis of DTC after surgical resection, ultrasound-guided fine-needle aspiration cytology (FNAC) can be performed, and the sensitivity can reach 70–80% (10), while the sensitivity of computed tomography (CT) can reach 94.5% (11). In recent years, combined diagnostic methods such as US+CT (12) and US+ Contrast-Enhanced Ultrasonography (CEUS) (13) have also been commonly used to diagnose the LNM of PTC. Among these studies, the preoperative diagnostic performance of different diagnostic methods for LNM of PTC has been evaluated and analyzed or only one or two diagnostic methods have been compared, but a thorough evaluation has not been performed. Since different diagnostic methods have different diagnostic efficacy, and the diagnostic efficacy of the same diagnostic method is different in different studies, we conducted a meta-analysis to evaluate the diagnostic performance of different diagnostic methods for LNM of PTC so as to obtain the optimal diagnostic protocol.

In this study, we summarized the available evidence, investigated and compared a variety of different diagnostic techniques through network meta-analysis and the use of two or more published direct comparative studies, and concluded the optimal diagnostic protocol for LNM of PTC through comprehensive analysis. The results of this study will provide more evidence-based data for the development of guidelines and will guide patients with LNM of PTC to use appropriate diagnostic methods for preoperative or postoperative evaluation.

## 2 Methods

### 2.1 Retrieval strategy

We used keywords such as “Thyroid Neoplasms”, “lymph node”, “Neoplasm Staging”, “Lymphatic Metastasis”, and”Elasticity Imaging Techniques” in PubMed, Embase, Web of Science, CNKI, and Wanfang databases for retrieval. In order to obtain more sufficient data, we also screened the references of the retrieved articles (**Table 1**).

**Table 1:** Search strategy.

No.	Retrieval type
#1	lymph node【Mesh】
#2	Neoplasm Staging【Mesh】
#3	Staging, Neoplasm【Title/Abstract】
#4	Tumor Staging【Title/Abstract】
#5	TNM Staging System【Title/Abstract】
#6	TNM Classifications【Title/Abstract】
#7	preoperative staging【Title/Abstract】
#8	Lymphatic Metastasis【Mesh】
#9	Lymphatic Metastases【Title/Abstract】
#10	Lymph Node Metastasis【Title/Abstract】
#11	Lymph Node Metastasis【Title/Abstract】
#12	Metastasis, Lymph Node【Title/Abstract】
#13	#1OR #2 OR #3 OR#4OR#5OR#6 OR #7 OR#8 OR#9OR #10 OR#11OR#12
#14	Thyroid Neoplasms【Mesh】
#15	Neoplasm, Thyroid【Title/Abstract】
#16	thyroid carcinoma【Title/Abstract】
#17	thyroid cancer【Title/Abstract】
#18	thyroid neoplasm【Title/Abstract】
#19	Cancer of the Thyroid【Title/Abstract】
#20	Thyroid Cancers【Title/Abstract】
#21	#14OR#15OR#16 OR #17 OR#18 OR#19OR #20
#22	ultrasound contrast【Title/Abstract】
#23	Elasticity Imaging Techniques【Mesh】
#24	Elastography【Title/Abstract】
#25	Elastograms【Title/Abstract】
#26	B-mode【Title/Abstract】
#27	Ultrasonography【Mesh】
#28	Diagnostic Ultrasound【Title/Abstract】
#29	Ultrasound Imaging【Title/Abstract】
#30	Ultrasonic Imaging【Title/Abstract】
#31	Ultrasonic Diagnosis【Title/Abstract】
#32	Ultrasonic Diagnosis【Title/Abstract】
#33	Ultrasound-Guided Fine-Needle aspiration【Title/Abstract】
#34	Magnetic Resonance Imaging【Mesh】
#35	MRI Scan【Title/Abstract】
#36	Positron Emission Tomography Computed Tomography【Mesh】
#37	PET-CT Scan【Title/Abstract】
#38	18F-FDG PET/CT【Title/Abstract】
#39	131I【Title/Abstract】
#40	IWBS【Title/Abstract】
#41	131I scintigraphy【Title/Abstract】
#42	#23 OR#24OR#25OR#26 OR #27 OR#28 OR#29OR #30 OR#31OR#32#33 OR#34OR#35OR#36 OR #37 OR#38 OR#39OR #40 OR#41
#43	#13 AND#21AND#42

#### 2.1.1 Inclusion and exclusion criteria

Inclusion criteria: ① Research subjects: PTC patients diagnosed with LNM. ② Study type: A randomized controlled study was conducted and two or more functional or non-invasive diagnostic methods should be included. ③ Gold standard: postoperative histopathology reports. ④Outcome indicators: It can reflect the sensitivity (SEN), specificity (SPE), positive predictive value (PPV), negative predictive value (NPV), and accuracy of various diagnostic methods for LNM in patients with PTC.

Exclusion criteria: ① There were less than 2 diagnostic methods in the study; ② There were no clear inclusion and exclusion criteria in the study; ③Reviews and lecture-type literature. ④ Literature for which the full text cannot be obtained.

### 2.2 Literature screening

Two researchers (Qiao-Li Li, Si-Rui Wang) read the titles and abstracts of the retrieved literature respectively. According to the inclusion and exclusion criteria established in this study, the literatures that did not meet the inclusion criteria were excluded, and the literatures that might meet the inclusion criteria and other related literatures were obtained and the relevant literatures were further intensively read. Articles with disagreements that were difficult to determine whether to be included were determined through discussion or consultation with a third party (Jun Li).

### 2.3 Quality evaluation of literature

For the RCT study, according to the method provided by the Cochrane Handbook, the research group adopted the risk of bias assessment tool of the Cochrane Collaboration (14) (RevMan v.5.3.5, Cochrane Collaboration, Oxford, UK), evaluated the methodological quality of the included studies from 6 aspects, and made the judgment of “yes” (low bias), “no” (high bias) and “unclear” (lack of relevant information or uncertainty of bias).

### 2.4 Data extraction

The data extracted in this study mainly included: (1) Characteristics of studies in the literature (author, publication time, country, study type, gold standard, etc.) (2) Subject characteristics (sample size, gender of patients, mean age or age range) (3) Effect indicators.

### 2.5 Statistical analysis

We grouped them according to different diagnostic methods and performed a network meta-analysis using the extracted diagnostic tools for the diagnostic performance of lymph node metastases.

We used Stata software (version-15.1) to perform the aggregation and analysis of NMA using Markov Chain Monte Carlo Subset Simulation in a Bayesian-based framework according to the instruction manual for Prisma NMA (15). We used the nodal method to quantify and demonstrate the agreement between direct and indirect comparisons. Through the calculation of the instructions in the Stata software and whether the P value was greater than 0.05, it was judged whether the consistency check was passed.

Network diagrams of different exercise interventions were presented and described using Stata software. In the resulting network diagram, each node represented a different diagnostic approach, and the lines connecting the nodes represented direct head-to-head comparisons between interventions. The size of each node and the width of the connecting lines were proportional to the number of studies.

The diagnostic performance of each diagnostic method was analyzed by the area under SUCRA, and the certainty that one method was superior to the other was measured. Although SUCRA could effectively express the percentage of diagnostic performance of each diagnostic method, there was a possibility that there was no actual clinical significance between the diagnostic methods. In order to detect whether there was publication bias in some studies, funnel plots were generated for each diagnostic efficacy, and symmetry criterion were used to check.

## 3 Results

### 3.1 Selection and characteristics of literature

**Figure 1:**
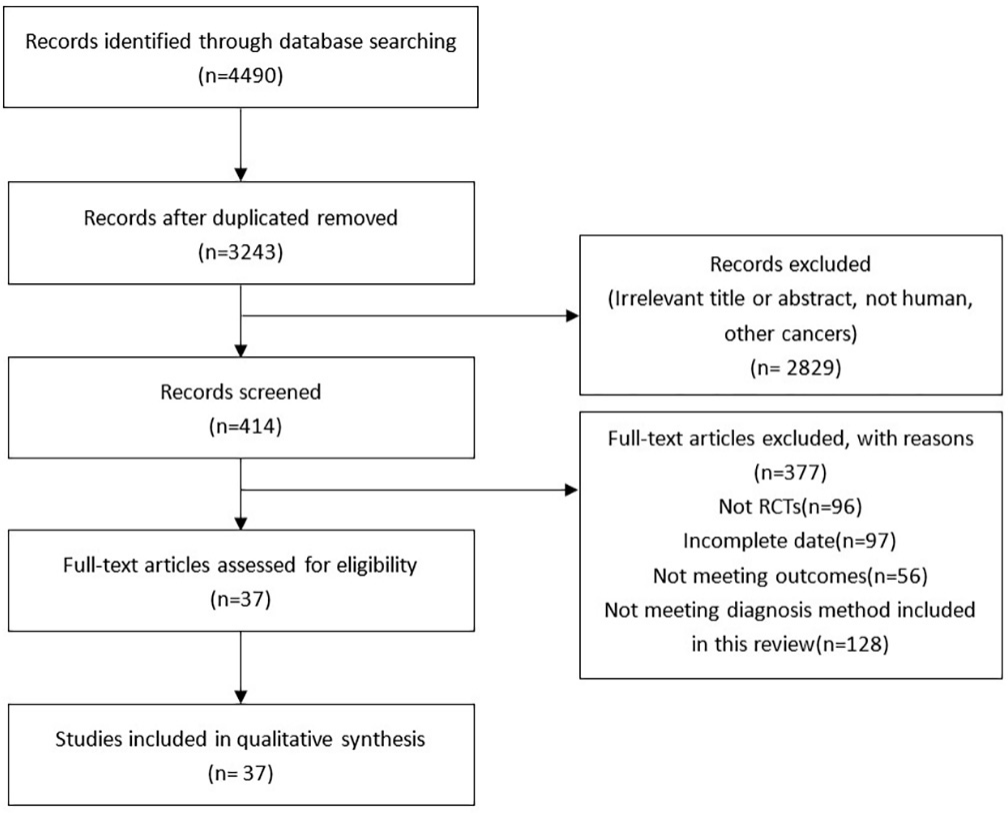
Flow diagram of literature selection.

A total of 4490 articles were extracted through literature search of the database and reference extraction, of which we excluded 1247 duplicate articles. Of the remaining 3243 articles, there were 2829 articles, 59 pathology reports, 699 review articles, 10 letters, and 72 meta-analyses and systematic reviews. 2061 articles not related to this study were excluded by review of the title and abstract. Among the remaining 414 articles, 376 articles failed to obtain the full text or failed to meet the inclusion criteria, and finally 38 articles were included, with a total of 6285 people. (**Figure 1**). The articles we included all fulfilled the requirement that the study population was patients with LNM of PTC and that their diagnoses were shown preoperatively or postoperatively by two or more diagnostic methods, such as US, CT, and MRI, and the data for direct comparison in the results were evaluated (**Table 2**).

**Table 2:** Characteristics of the studies included in the meta-analysis.

First Author	Year	Country	Design	NO.of patients	Gender(M/F)	Histology	LN level	Diagnostic method	NO.of patients	Gender(M/F)	Histology
**Artür Salmaslıoğlu (** 16 **)**	2010	Turkey	RCT	225	60/165	225PTC	ALL	③	④		
**Ari Chong (** 17 **)**	2017	Korea	RCT	85	71/14	85PTC	C, L	⑦	⑥		
**Byung Hyun Byun (** 18 **)**	2012	Korea	RCT	200	41/255	200PTC	ALL, C, L	②	⑥		
**C. De Crea (** 19 **)**	2014	Italy	RCT	38	16/22	38PTC	C	④	③		
**Cesur Samanci (** 20 **)**	2019	Turkey	RCT	32	13/19	26PTC, 6FTC	ALL	⑥	⑨		
**D.W.Lee (** 21 **)**	2013	Korea	RCT	252	45/207	252PTC	C, L	②	⑦	②+⑦	
**David Lesnik (** 22 **)**	2013	USA	RCT	162	NA	162PTC	C, L	②	⑦	②+⑦	⑥
**Enke Baldini (** 23 **)**	2013	Italy	RCT	28	7/21	21PTC, 2MTC,2ATC	ALL	③	④		
**Eunhee Kim (** 13 **)**	2008	Korea	RCT	165	25/140	165PCT	ALL, C, L	②	⑦	②+⑦	
**Eun NL (** 24 **)**	2017	Korea	RCT	302	76/226	302PTC	ALL	③	③+④		
**Farzana Alam (** 25 **)**	2008	Japan	RCT	37	12/25	37PTC	ALL	②	①		
**Han Sin Jeong (** 26 **)**	2006	Korea	RCT	26	7/19	26PTC	ALL	⑥	②		
**Harry S. Hwang (** 27 **)**	2009	USA	RCT	42	NA	42PTC	ALL	⑥	②		
**Jasna Mihailovic (** 28 **)**	2010	Serbia	RCT	40	15/25	39PTC, 1FTC	ALL	⑨	⑤		
**Jeon SJ (** 29 **)**	2009	Korea	RCT	47	NA	47PTC	ALL	③	④	③+④	
**Ji Soo Choi (** 30 **)**	2009	Korea	RCT	299	44/255	186PTMC, 113PTC	C, L	②	⑦	②+⑦	
**Ji-Hoon Jung (** 31 **)**	2015	Korea	RCT	193	25/168	151PTMC, 42PTC	ALL	⑥	②		
**Jia Zhan (** 32 **)**	2019	China	RCT	56	16/40	56PTC	C	②	⑧		
**Johann-Martin Hempel (** 33 **)**	2016	Germany	RCT	46	27/19	16FCT, 29PTC	ALL	⑥	⑨		
**Jun Ho Lee (** 21 **)**	2015	Korea	RCT	78	26/52	78PTC	ALL	③	④		
**Kim MJ (** 34 **)**	2012	Korea	PRO	68	16/52	68PTC	ALL	④	③		
**Khadra (** 35 **)**	2018	USA	RCT	138	31/106	138PTC	ALL	③	④	③+④	
**Lei Chen (** 13 **)**	2019	China	RCT	46	6/40	46PTC	ALL	②	⑧	②+ ⑧	
**Li J (** 36 **)**	2016	China	RCT	124	23/101	124PTC	ALL	③	④	③+④	
**Shi JH (** 37 **)**	2015	China	RCT	148	NA	148PTC	ALL	④	③	③+④	
**So Yeon Yang (** 38 **)**	2019	Korea	RCT	453	108/345	453PTC	ALL	②	⑦	②+⑦	
**Sohn YM (** 39 **)**	2012	Korea	RCT	92	NA	92PTC	ALL	④	③		
**Suh YJ (** 40 **)**	2013	Korea	RCT	43	NA	43PTC	ALL	④	③	③+④	
**Tong Tong Liu (** 41 **)**	2018	China	RCT	75	20/55	75PTC	C	②	①	②+①	
**Yanfang Wang (** 42 **)**	2021	China	RCT	120	37/83	120PTC,165PTMC	C	②	⑧		
**Ying Liu (** 43 **)**	2021	China	RCT	600	211/389	600PTC	C	②	⑦	②+⑦	
**Ying Wei (** 44 **)**	2020	China	RCT	24	14/10	24PTC	ALL	②	⑧		
**Yoon Jung Choi (** 45 **)**	2010	Korea	RCT	589	121/468	589PTC	C, L	②	⑦		
**Young Lan Seo (** 12 **)**	2012	Korea	RCT	20	4/16	19PTC 1PTMC	ALL	⑥	②	⑦	
**Yurong Hong (** 46 **)**	2017	China	RCT	573	148/425	162PTC	All	②	⑧	②+⑧	
**Zeming Liu (** 47 **)**	2014	China	RCT	70	NA	70DTC	C, L	⑨	②		
**Zhao H (** 48 **)**	2017	China	PRO	189	NA	189PTC	ALL	④	③	③+④	

Lymph node level (LN level); All: All cervical LN; C: Central LN; L: Lateral LN.

PTC, papillary thyroid carcinoma; PTMC, papillary thyroid microcarcinoma; FTC, Follicular thyroid carcinoma; MTC, Medullary thyroid cancer;

① SE-US, strain elastography; ② US, Ultrasound.; ③ FNAC, fine-needle aspiration cytology; ④ FNA-Tg, fine-needle aspiration thyroglobulin; ⑤ IWBS, 131I whole-body; ⑥ 18F-FDG PET/CT, F-18 fluorodeoxyglucose positron emission tomography/computed tomography; ⑦ CT, Computed tomography; ⑧ CEUS, Contrast-Enhanced Ultrasonography; ⑨ MRI, Magnatic Resonance Imaging.

### 3.2 Quality assessment and publication bias

**Figure 2:**
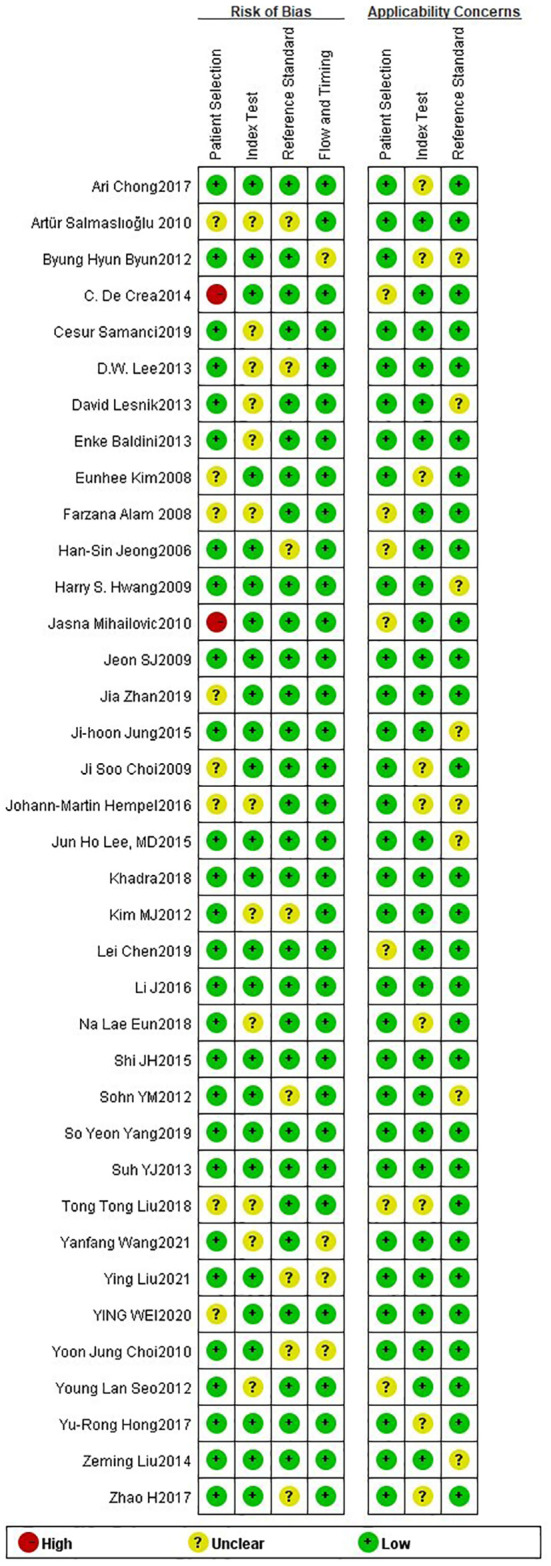
Bias risk of the included studies (QUADAS 2 criteria).

**Figure 3:**
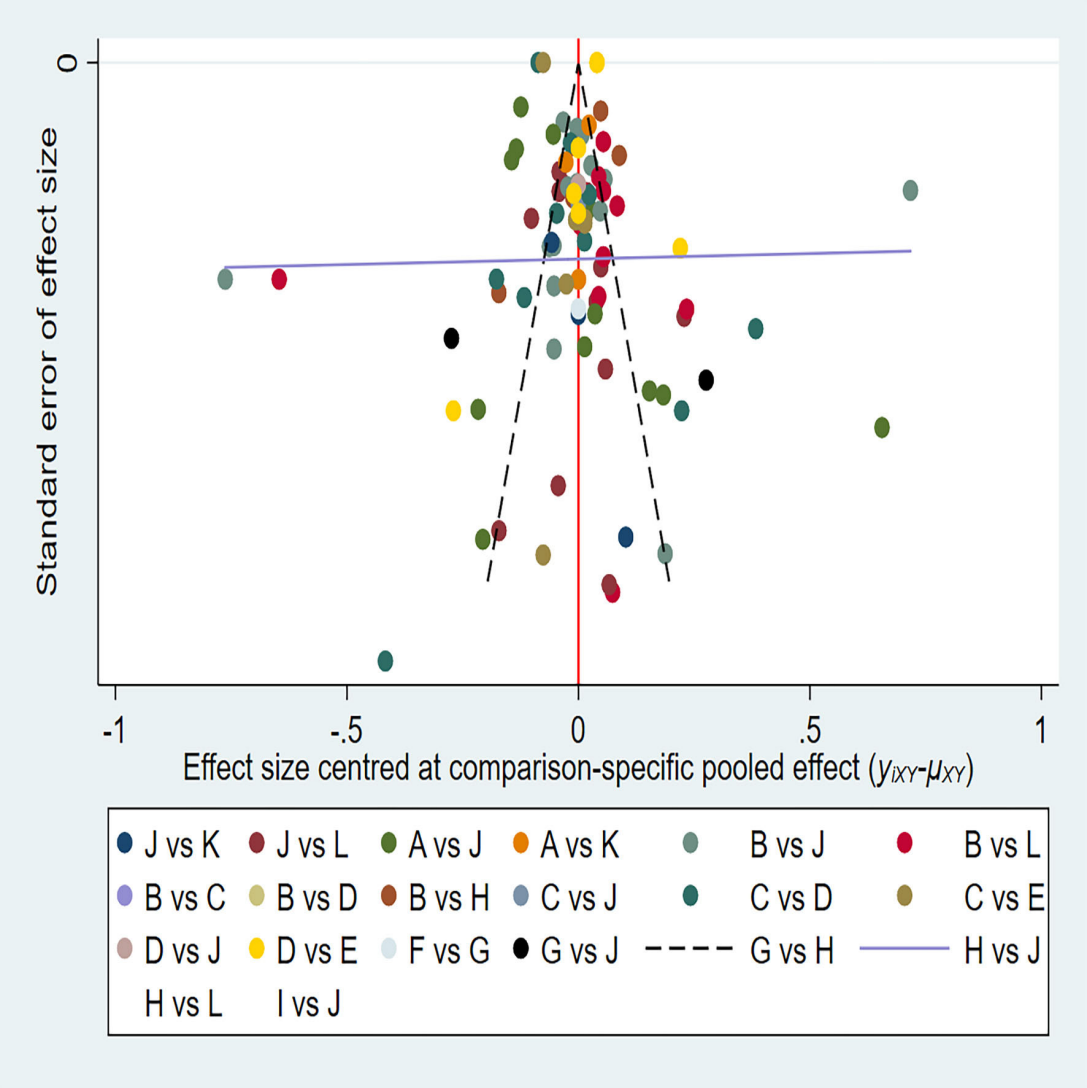
**A** = CEUS, **B** = CT, **C** = FNA-Tg, > **D** = FNAC, **E** = FNAC+FNA-Tg, **F** = IWBC, **G** = MRI, **H** = PET/CT, **I** = SE-US, **J** = US, **K** = US+CEUS, **L** = US+CT.

The 38 articles we included (11–13, 15–45, 47, 49) included 36 retrospective studies and 2 prospective studies. We performed a network meta-analysis using STATA 15.0 (14), and 38 articles were assessed for quality, risk of bias and applicability issues using QUADAS-2 (50). The overall quality of the articles was satisfactory, but there may be potential bias in personnel selection. Among the 38 articles, 8 articles had unclear risk of bias, and 2 articles had high risk. In terms of index testing, because some doctors did not strictly implement blinding in the processing of results, there were 12 literatures with unclear risk of bias. In terms of reference standard bias, there were 6 articles with unclear risk of bias, because it was not indicated whether there was an appropriate time interval between the trial to be evaluated and the gold standard. Two articles had unclear risk of bias with respect to follow-up time. In terms of applicability, all literatures showed no high risk of bias in patient selection, index test, and reference standard. (**Figure 2**) The authors' assessment of each domain for included study. This study used funnel plots to detect possible publication bias, and the results showed that the distribution of funnel plots was roughly symmetric, suggesting that there was no publication bias or other bias in the study (**Figure 3**).

### 3.3 Pairwise meta-analysis

**Figure 4:**
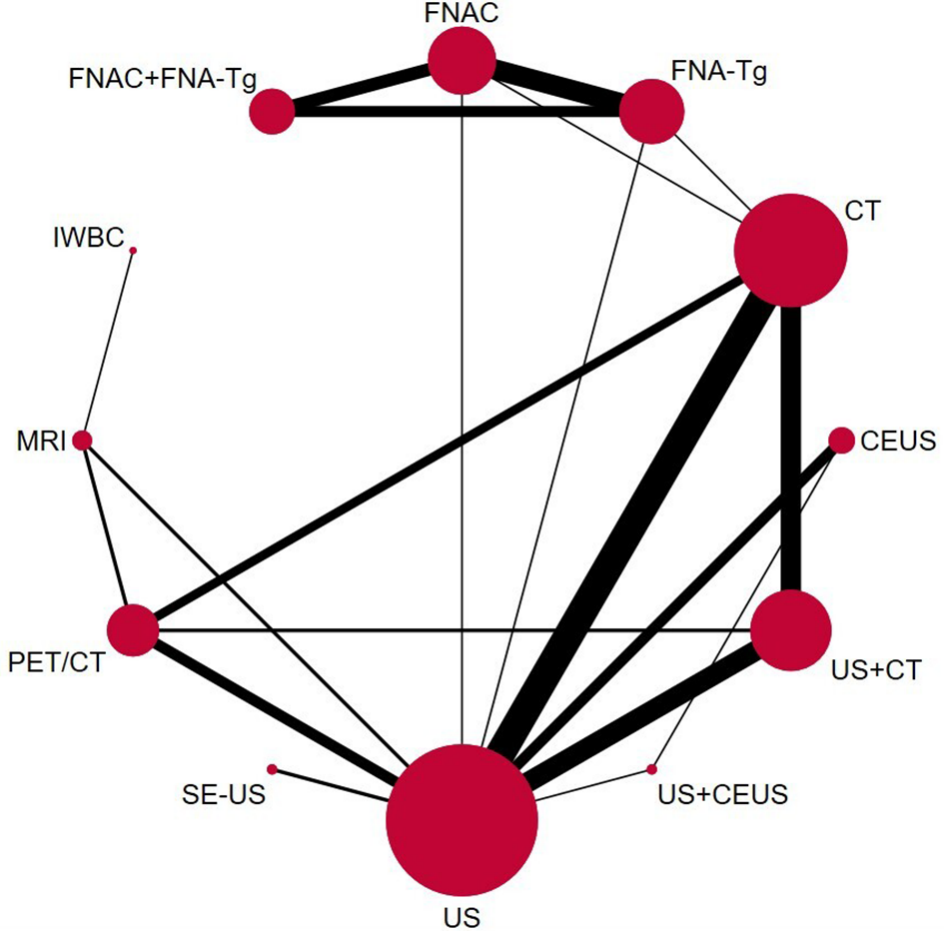
Network Mate-Analysis Figure.

Through the results of SEN, SPE, PPV, NPV, and Accuracy of various diagnostic methods, a network meta-analysis graph can be made (**Figure 4**), in which the gridlines in the analysis diagram represented a direct comparison between the two groups of diagnostic methods, the thickness of the gridlines represented the number of articles included, and the size of the solid dots at both ends of the gridlines represented the sample size. Of the 38 studies compared, 12 were direct comparisons and 8 were indirect comparisons. We performed a direct pair-wise comparison of SEN, SPE, PPV, NPV, and Accuracy for each method of diagnosing LNM of PTC. Meta-analyses of the results can be used to make direct comparisons. There were 3 studies comparing MRI with PET/CT, 2 studies comparing CT with FNA-Tg, 16 studies comparing US with CT, 6 studies comparing FNAC with FNA-Tg, 3 studies comparing US+CEUS with US, 5 studies comparing CT with PET/CT, 7 studies comparing US with CEUS, 2 studies comparing CT with FNAC, 2 studies comparing US with FNAC, 9 studies comparing US+CT with US, 6 studies comparing US with PET/CT, 3 studies comparing US+CEUS with CEUS, 9 studies comparing US+CT with CT, 2 studies comparing MRI with IWBC, 3 studies comparing US with SE-US, 3 studies comparing FNA-Tg with FNA-Tg+FNAC, and 3 studies comparing FNAC with FNA-Tg+FNAC. As shown in the figure.

### 3.4 Network meta-analysis

The OR values and 95% confidence intervals (CI) of SEN, SPE, PPV, NPV, and Accuracy measured by different diagnostic methods for LNM of PTC were statistically analyzed, and the statistical differences were judged by calculation.

#### 3.4.1 SEN

**Figure 5:**
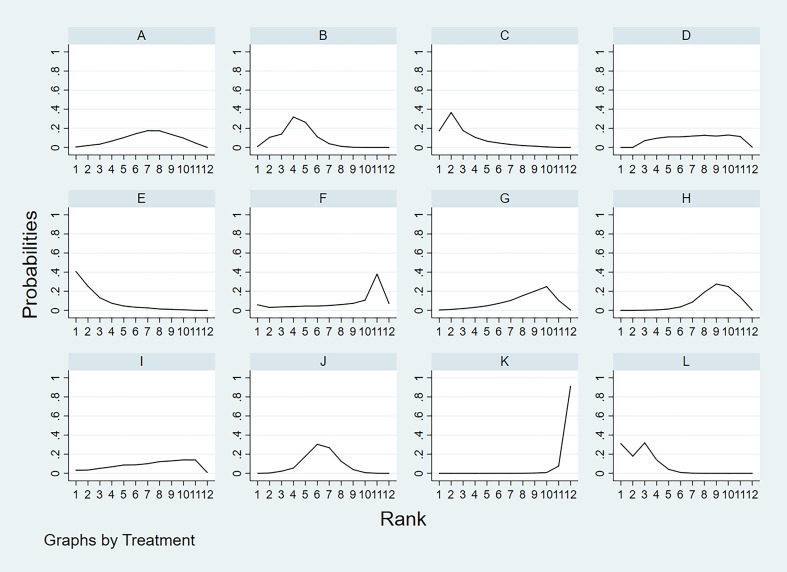
**A** = CEUS, **B** = CT, **C** = FNA-Tg, > **D** = FNAC, **E** = FNAC+FNA-Tg, **F** = IWBC, **G** = MRI, **H** = PET/CT, **I** = SE-US, **J** = US, **K** = US+CEUS, **L** = US+CT.

**Figure 6:**
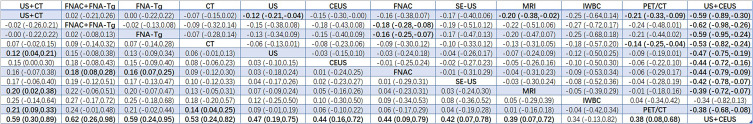
League table on SEN.

According to the area under the cumulative ranking curve (SUCRA), the SEN of different diagnostic methods for lymph node metastasis can be obtained. The descending order was as follows: US+CT(86.8)>FNAC+FNA-Tg(86.6) >FNA-Tg(81.8)>CT(70.2)>US(51.8)>CEUS(44.5)>FNAC(42.5)>SE-US(42.2)>MRI(33.4)>IWBC(31.7)>PET/CT(27.5)>US+CEU (1.0). Among them, US+CT (OR=0.59, 95%CrI:(0.30,0.89)) and FNAC+FNA-Tg[OR=0.62,95%CI:(0.26,0.98)] ranked first and second, respectively, and had significant advantages compared with other diagnostic methods. There was significant heterogeneity between MRI and US, PET/CT in terms of sensitivity (P<0.05). The probability ranking of diagnosis methods to SEN was ranked first by US+CT in the SUCRA (SUCRA:86.8% as shown in **Figure 5**). A comparison between the two different diagnosis methods was shown in (**Figure 6**).

#### 3.4.2 SPE

**Figure 7:**
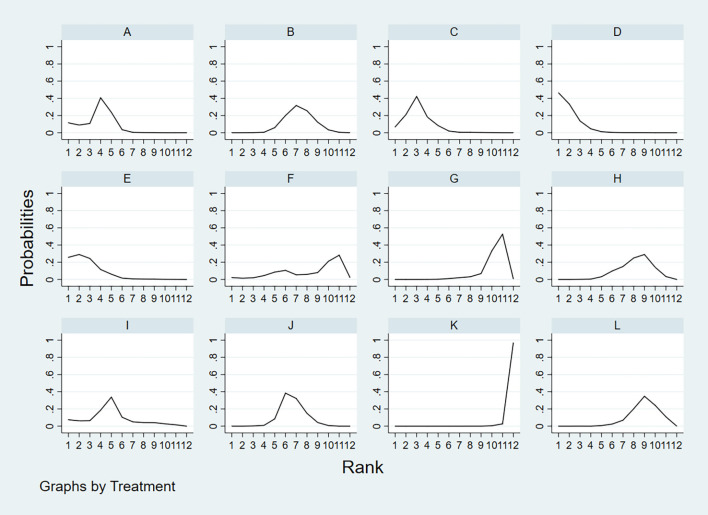
**A** = CEUS, **B** = CT, **C** = FNA-Tg, > **D** = FNAC, **E** = FNAC+FNA-Tg, **F** = IWBC, **G** = MRI, **H** = PET/CT, **I** = SE-US, **J** = US, **K** = US+CEUS, **L** = US+CT.

**Figure 8:**
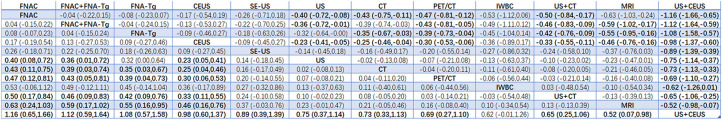
League table on SPE.

According to the area under the cumulative ranking curve (SUCRA), the SPE of different diagnostic methods for lymph node metastasis can be obtained. The descending order was as follows: FNAC(92.4)>FNAC+FNA-Tg(85.8)>FNA-Tg(80.5)>CEUS(75.4)>SE-US(64.4)>US(48.5)>CT(42.9)>PET/CT(34.5)>IWBC(32.6)>US+CT(27.1)>MRI(15.5)>US+CEUS(0.4). The analysis of the results showed that compared with US+CEUS, FNAC[OR=1.16,95%CI:(0.65,1.66)] and FNAC+FNA-Tg[OR=1.12,95%CI:(0.59,1.64)] had significant advantages. In terms of specificity (P<0.05), there was significant heterogeneity in the comparison between FNAC and US, FNA-Tg and US, and FNAC and CT. The probability ranking of diagnosis methods to SPE was ranked first by FNAC in the SUCRA (SUCRA:92.4% as shown in **Figure 7**). A comparison between the two different diagnosis methods was shown in **Figure 8**.

#### 3.4.3 PPV

**Figure 9:**
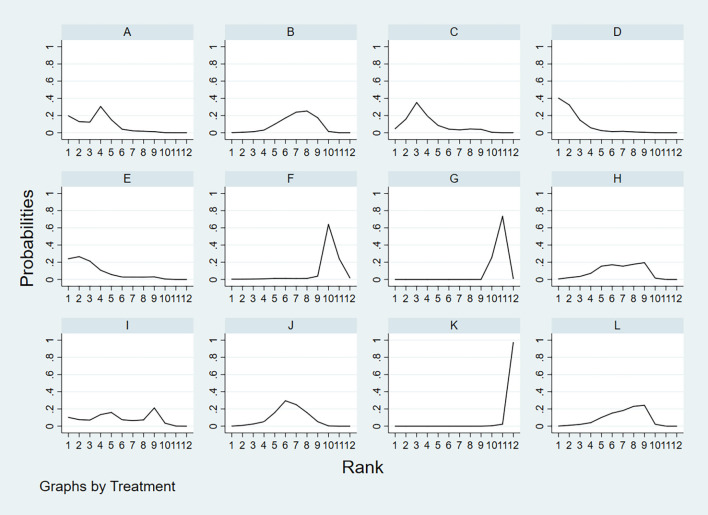
**A** = CEUS, **B** = CT, **C** = FNA-Tg, > **D** = FNAC, **E** = FNAC+FNA-Tg, **F** = IWBC, **G** = MRI, **H** = PET/CT, **I** = SE-US, **J** = US, **K** = US+CEUS, **L** = US+CT.

**Figure 10:**
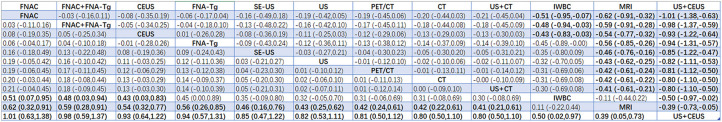
League table on PPV.

According to the area under the cumulative ranking curve (SUCRA), the PPV of different diagnostic methods for lymph node metastasis can be obtained. The descending order was as follows: FNAC(89.4)>FNAC+FNA-Tg(81.6)>CEUS(77.6)>FNA-Tg(73.9)>SE-US(58.7)>US(51.3)>PET/CT(48.7)>CT(44.5)>US+CT(43.9)>IWBC(18.6)>MRI(11.3)>US+CEUS(0.3). The analysis of the results showed that compared with US+CEUS, FNAC[OR=1.01,95%CI:(0.63,1.38)] and FNAC+FNA-Tg[OR=0.98,95%CI:(0.59,1.37)] had significant advantages. There was no significant difference in specificity (P<0.05). The probability ranking of diagnosis methods to PPV was ranked first by FNAC in the SUCRA (SUCRA: 89.4% as shown in **Figures 9**). A comparison between the two different diagnosis methods was shown in **Figure 10**.

#### 3.4.4 NPV

**Figure 11:**
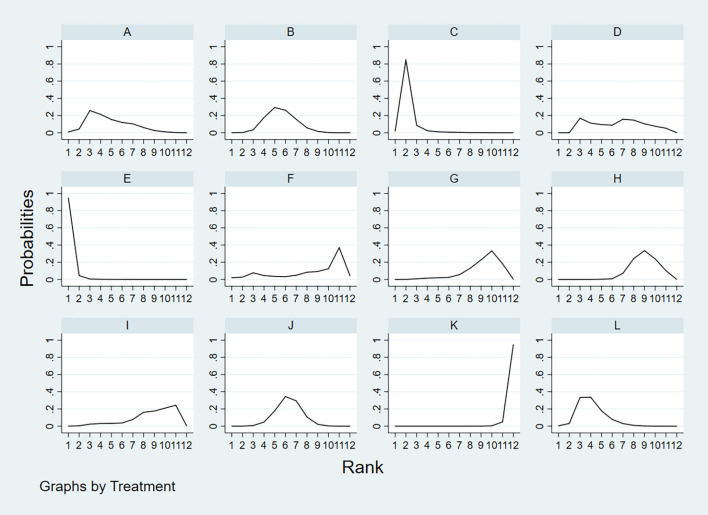
**A** = CEUS, **B** = CT, **C** = FNA-Tg, > **D** = FNAC, **E** = FNAC+FNA-Tg, **F** = IWBC, **G** = MRI, **H** = PET/CT, **I** = SE-US, **J** = US, **K** = US+CEUS, **L** = US+CT.

**Figure 12:**
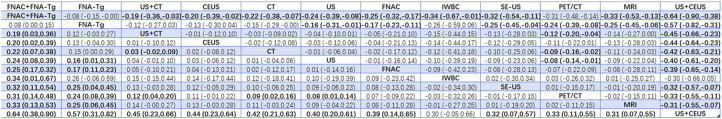
League table on NPV.

According to the area under the cumulative ranking curve (SUCRA), the NPV of different diagnostic methods for lymph node metastasis can be obtained. The descending order was as follows: FNAC+FNA-Tg(99.4)>FNA-Tg(89.0)>US+CT(72.2)>CEUS(65.6)>CT(58.5)>US(52.0)>FNAC(49.9)>IWBC(31.4)>SE-US(28.8)>PET/CT(27.1)>MRI(25.6)>US+CEUS(0.6). The analysis of the results showed that compared with US+CEUS, FNAC+FNA-Tg (OR=0.64, 95%CrI: (0.38, 0.90) and FNA-Tg [OR=0.57, 95%CI: (0.31, 0.82)] had significant advantages. In terms of specificity (P<0.05), there was significant heterogeneity between MRI and US, PET/CT. The probability ranking of diagnosis methods to NPV was ranked first by FNAC+FNA-Tg in the SUCRA (SUCRA: 99.4% as shown in **Figures 11**). A comparison between the two different interventions was shown in **Figure 12**.

#### 3.4.5 Accuracy

**Figure 13:**
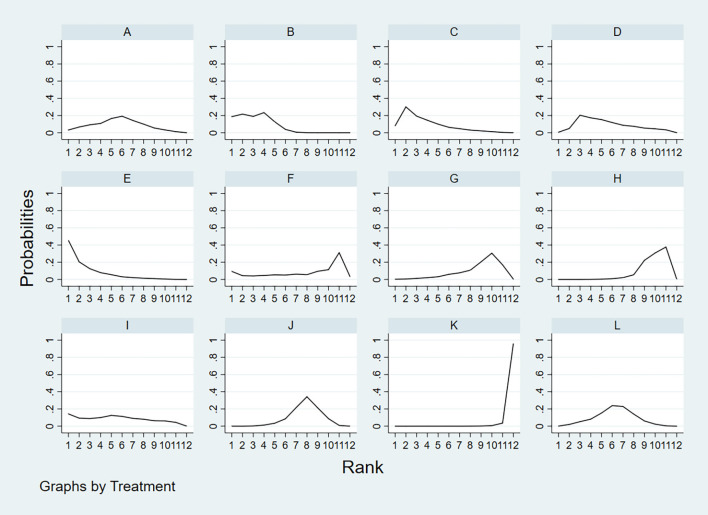
**A** = CEUS, **B** = CT, **C** = FNA-Tg, > **D** = FNAC, **E** = FNAC+FNA-Tg, **F** = IWBC, **G** = MRI, **H** = PET/CT, **I** = SE-US, **J** = US, **K** = US+CEUS, **L** = US+CT.

**Figure 14:**
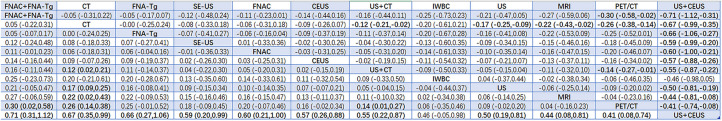
League table on ACC.

According to the area under the cumulative ranking curve (SUCRA), the Accuracy of different diagnostic methods for lymph node metastasis can be obtained. The descending order was as follows: FNAC+FNA-Tg(99.4)>CT(81.6)>FNA-Tg(76.0)>SE-US(61.6)>FNAC(59.8)>CEUS(57.8)>US+CT(52.4)>IWBC(39.0)>US(37.2)>MRI(28.6)>PET/CT(18.7)>US+CEUS(0.5). The analysis of the results showed that compared with US+CEUS, FNAC+FNA-Tg[OR=0.71,95%CI:(0.31,1.12)] and FNA-Tg[OR=0.67,95%CI:(0.35,0.99)] had significant advantages. In terms of specificity (P<0.05), there was significant heterogeneity between CT and US+CT, PET/CT and US, and PET/CT and US+CT. The probability ranking of diagnosis methods to ACC was ranked first by FNAC+FNA-Tg in the SUCRA (SUCRA: 99.4% as shown in **Figure 13**). A comparison between the two different diagnosis methods was shown in **Figure 14**.

## 4 Discussion

This meta-analysis aimed to evaluate the diagnostic performance of multiple diagnostic methods for predicting LNM of PTC. A total of 38 studies with 6285 patients were summarized and analyzed. Among the 12 diagnostic methods, we found that the combined diagnosis of CT+US and FNAC+FNA-Tg was the most effective diagnostic method for the recurrence and metastasis of LNM of PTC. A total of 6 articles were included in the comparison of US and CT, and the diagnostic performance of CT and US for LNM of PTC, such as sensitivity and specificity, were statistically analyzed. By analyzing the data we found that for the sensitivity of US and CT, the values of SUCRA were 51.8 and 70.2, respectively, while the value for the combined diagnosis of US+CT was 86.8. David (49) et al. found that the accuracy of US may be affected due to the difficulty in distinguishing between the metastatic lymph nodes immediately adjacent to the thyroid and the thyroid itself. In addition, some normal anatomical structures cause distinct acoustic shadows that effectively obscure the area behind them, including air-filled structures such as the larynx and trachea, as well as the clavicle and jawbone, areas deep in the sternum. This greatly limits the assessment of the mediastinal and retropharyngeal regions by ultrasound, regardless of the operator’s interest and expertise. However, all of these areas are commonly seen on CT, and CT scans in the central neck are twice as sensitive as US. Zhao H et al. (51) found that CT had higher sensitivity in the central region and throughout the lateral regions. However, ultrasound had a higher specificity in the central and whole lateral regions. CT can better reduce the missed diagnosis rate, while US can better avoid misdiagnosis. Therefore, this suggested that clinicians should consider the combination of CT and US when diagnosing LNM of PTC. This was consistent with our results that the combined diagnosis method of CT+US could more accurately diagnose LNM for better clinical decision-making.

This time, a variety of diagnostic methods for lymph node metastasis of thyroid cancer were compared. As far as we know, although some authors have published meta-analyses of two or more diagnostic methods for lymph node metastasis of thyroid cancer, our network meta-analysis was the first to comprehensively analyze the currently known diagnostic methods. As far as the retrieved literature is concerned, there are obviously more studies on US and CT than those on other diagnostic methods, indicating that these two diagnostic techniques are easier for statistical analysis through direct comparison, and at the same time indicating that US and CT are more widely accepted by everyone, while there are few studies that directly compare CEUS with FNA-Tg. It is recommended to carry out more original research on the comparative analysis of the diagnostic performance of these two diagnostic methods. At the same time, there are many studies comparing CT, US, and PET/CT in their diagnostic performance for lymph node metastasis of thyroid cancer, which also shows that these three studies have obvious comparative value (52). Through analysis, we found that such invasive diagnostic methods such as the combined application of FNAC,FNA-Tg and FNAC+FNA-Tg had better diagnostic performance. Although non-invasive diagnostic methods were more popular, except for the combined application of US+CT (19, 33), the diagnostic performance of other diagnostic methods was not ideal.

PTC is generally considered to be a tumor with a low probability of metastasis and recurrence. In some patients, PTC also occurs with lymph node metastasis and local recurrence (53). Studies have shown that tumor size, extrathyroidal extension and vascular invasion are independent risk factors for lymph node metastasis (54). However, extrathyroid extension and vascular infiltration are not helpful to surgeons undergoing surgery, and imaging features such as tumor size and shape can effectively improve the ability of preoperative diagnosis of PTC lymph node metastasis (55).

At present, most hospitals mostly use this non-invasive method of US and CT for preoperative or postoperative evaluation and examination of patients with LNM of PTC. However, US also has its own limitations. US is far from satisfactory in sensitivity and accuracy for lymph node metastasis. It cannot observe deeper tissues or lymph node metastasis, and the accuracy of the result often depends on the experience of the diagnostician, etc. CT is generally considered to be a diagnostic method that plays a key role in the diagnosis of lymph node metastasis of thyroid cancer, especially in the evaluation of deeper tissues (56). CT is slightly inferior to US in evaluating the diagnostic value of central and lateral cervical lymph nodes. Therefore, the combination of CT and US has been evaluated in 11 studies. In our study, regarding the sensitivity to thyroid nodules and lymph node infiltration, we found that the sensitivity of US+CT in the diagnosis of thyroid nodules and lymph node infiltration was superior to the sensitivity of FNAC and FNA-Tg alone.

FNAC was highly specific in the analysis, and Suh YJ et al. (36, 37, 39) found that the threshold values of FNA-Tg established in different experiments were different. If the threshold value of FNA-Tg was specified as 1ng/mL, and levels greater than 1ng/mL were accepted as the surgical indication, then NPV was 0%. Non-metastatic lymph nodes ranged in value from 2–32 ng/mL. The SPE, PPV, NPV and Accuracy of FNA-Tg were significantly lower than those of FNAC. When the critical value of FNA-Tg was 28.5ng/ml, the SPE, PPV, NPV and Accuracy were significantly higher than those of FNAC. Therefore, further experiments are required to determine the optimal critical value of FNA-Tg. Based on our analysis, the diagnostic performance of the combination of FNAC+FNA-Tg was significantly superior to that of FNAC and FNA-Tg alone. In the combination application of FNAC and FNA-Tg, the values of PPV, NPV and Accuracy in SUCRA were 81.6,99.4 and 86.8 respectively, which had obvious advantages in the diagnosis of LNM of PTC and also proved that the combination application of FNAC and FNA-Tg was one of the necessary diagnostic methods for accurate diagnosis of benign and malignant lymph node metastasis.

Although the combined diagnosis has higher diagnostic performance than the single diagnosis method, the price of the same part of the examination will be correspondingly increased, and the economic burden of different examination methods for patients is different. In China, some routine examinations such as CT,MRI,US,PET/CT are within the range of 100–800 RMB (57), which will not cause great economic pressure for most families. Moreover, the combination diagnosis of CT and US can also obtain relatively accurate results. However, the prices of FNAC and FNA-Tg are about 2000RMB, which is much more expensive compared with conventional examinations. Moreover, most of the patients undergoing such examinations are patients with LNM of PTC, who need to go to the hospital for regular reexamination in addition to the expenses required for daily treatment, so that they need to bear a heavier economic burden. Therefore, during the examination, we should not blindly pursue more accurate diagnosis performance of the disease, but we should be more concerned about their demands and the economic pressure they can bear from the perspective of patients. In the United States, the average cost of these routine imaging examinations in upper-level academic hospitals ranges from $686.13 to $1,390.12. The reason for the higher price in the United States may be that most residents have purchased health insurance, and they can only go to the designated hospitals with medical insurance for examinations, resulting in that they cannot go to the hospitals with lower costs for relevant examinations. At the same time, the price of related examinations is less transparent. Hospitals with lower prices may be stimulated by higher-priced hospitals, leading to price increases. All these have correspondingly increased the economic burden of consumers.

### 4.1 Limitation

At present, there are many diagnostic methods for lymph node metastasis of papillary thyroid carcinoma, but there are still differences in these diagnostic values, and further analysis and standardization are needed. (1) Compared with previous meta-analyses, we have included more diagnostic methods, but few articles have included some diagnostic methods, and their reliability required more experiments to verify. (2) It was found in the study that the results of direct comparison between interventions were less, and most of them were the results of indirect comparison. This would also affect the final quality evaluation results, which meant that further more direct comparison between different diagnostic methods was needed in the study. (3) Some results showed obvious heterogeneity in our study, but no source of heterogeneity was found after heterogeneity analysis. In view of the above shortcomings, it is recommended that the readers reasonably refer to and select the diagnostic methods in this study based on the clinical practice and actual results.

## 5 Results

In conclusion, we believe that the combined application of CT+US and FNAC+FNA-Tg is the optimal solution for the diagnosis of lymph node metastasis in papillary thyroid carcinoma, but further prospective studies on its cost-effectiveness and clinical diagnostic performance are still needed.

## Author contributions

Study concept and design: S-RW, Q-LL. Acquisition of data: Q-LL, MC, TS, S-RW, FT. Analysis and interpretation of data: C-LC, S-RW, FT. Drafting of the manuscript: S-RW, FT. Critical revision of themanuscript for important intellectual content: JL. Approval of the final manuscript: JL, L-NS, TS, C-LC, S-RW, FT. Study supervision: JL.

## Funding

1.Supported by the Non-profit Central Research Institute Fund of Chinese Academy of Medical Sciences (2020-PT330-003) 2.Supported by Open Research Fund of NHC Key Laboratory of Prevention and Treatment of Central Asia High Incidence Diseases 3.Key science and technology Project of Xinjiang Corps (2019DB012).

## Conflict of interest

The authors declare that the research was conducted in the absence of any commercial or financial relationships that could be construed as a potential conflict of interest.

## Publisher’s note

All claims expressed in this article are solely those of the authors and do not necessarily represent those of their affiliated organizations, or those of the publisher, the editors and the reviewers. Any product that may be evaluated in this article, or claim that may be made by its manufacturer, is not guaranteed or endorsed by the publisher.

## Abbreviations

PTC, papillary thyroid carcinoma; PTMC, papillary thyroid microcarcinoma; FTC, Follicular thyroid carcinoma; MTC, Medullary thyroid cancer; SE-US,strain elastography; FNAC, ultrasound-guided fine-needle aspiration cytology; FNA-Tg, fine-needle aspiration thyroglobulin; IWBS,131I whole-body; 18F-FDG PET/CT, F-18 fluorodeoxy glucose positron emission tomography/computed tomography; CT, Computed tomography; US, Ultrasound; CEUS, Contrast-Enhanced Ultrasonography; MRI,Magnetic Resonancediffusion Tension Imaging; SEN, Sensitivity; SPE, Specificity; PPV, positive predictive value; NPV, negative predictive value.

## References

[1] Vaccarella S Lortet-Tieulent J Colombet M Davies L CA S Schüz J. Global patterns and trends in incidence and mortality of thyroid cancer in children. Lancet Diabetes Endocrinol (2021) 9(3):144–52 144–52. 10.1016/S2213-8587(20)30401-0 33482107

[2] Olaleye O Ekrikpo U Moorthy R Lyne O Wiseberg J Black M. Increasing incidence of differentiated thyroid cancer in south East England: 1987-2006. Eur Arch Otorhinolaryngol (2011) 268(6):899–906. doi: 10.1007/s00405-010-1416-7 21072529

[3] Lim H Devesa SS Sosa JA Check D Kitahara CM. Trends in thyroid cancer incidence and mortality in the united states, 1974-2013. Jama (2017) 317(13):1338–48. doi: 10.1001/jama.2017.2719 PMC8216772 28362912

[4] Nikiforov YE Seethala RR Tallini G Baloch ZW Basolo F Thompson LD. Nomenclature revision for encapsulated follicular variant of papillary thyroid carcinoma: A paradigm shift to reduce overtreatment of indolent tumors. JAMA Oncol (2016) 2(8):1023–9. doi: 10.1001/jamaoncol.2016.0386 PMC5539411 27078145

[5] Lamartina L Grani G Durante C Filetti S. Recent advances in managing differentiated thyroid cancer. F1000Res (2018) 7:86. doi: 10.12688/f1000research.12811.1 29399330 PMC5773927

[6] Wunderbaldinger P Harisinghani MG Hahn PF Daniels GH Turetschek K Simeone J. Cystic lymph node metastases in papillary thyroid carcinoma. AJR Am J Roentgenol (2002) 178(3):693–7. doi: 10.2214/ajr.178.3.1780693 11856700

[7] Li F Pan D He Y Wu Y Peng J Li J. Using ultrasound features and radiomics analysis to predict lymph node metastasis in patients with thyroid cancer. BMC Surg (2020) 20(1):315. doi: 10.1186/s12893-020-00974-7 33276765 PMC7716434

[8] Chasen NN Wang JR Gan Q Ahmed S. Imaging of cervical lymph nodes in thyroid cancer: Ultrasound and computed tomography. Neuroimaging Clin N Am (2021) 31(3):313–26. doi: 10.1016/j.nic.2021.04.002 34243866

[9] Schlumberger MJ Torlantano M. Papillary and follicular thyroid carcinoma. Baillieres Best Pract Res Clin Endocrinol Metab (2000) 14(4):601–13. doi: 10.1053/beem.2000.0105 11289737

[10] Grani G Fumarola A. Thyroglobulin in lymph node fine-needle aspiration washout: a systematic review and meta-analysis of diagnostic accuracy. J Clin Endocrinol Metab (2014) 99(6):1970–82. doi: 10.1210/jc.2014-1098 24617715

[11] Seo YL Yoon DY Baek S Ku YJ Rho YS Eun-Jae Chung E-J. Detection of neck recurrence in patients with differentiated thyroid cancer: comparison of ultrasound, contrast-enhanced CT and f-18-FDG PET/CT using surgical pathology as a reference standard: (ultrasound vs. CT vs. f-18-FDG PET/CT in recurrent thyroid cancer). Eur Radiol (2012) 22(10):2246–54. 10.1007/s00330-012-2470-x 22562091

[12] Eunhee K Park J Son K Kim J-H Jeon SJ Na DG. Preoperative diagnosis of cervical metastatic lymph nodes in papillary thyroid carcinoma: Comparison of ultrasound, computed tomography, and combined ultrasound with computed tomography. Thyroid (2008) 18(4):411–8. 10.1089/thy.2007.0269 18358074

[13] Chen L Chen L Liu J Wang B Zhang H. Value of qualitative and quantitative contrast-enhanced ultrasound analysis in preoperative diagnosis of cervical lymph node metastasis from papillary thyroid carcinoma. J Ultrasound Med (2020) 39(1):73–81. doi: 10.1002/jum.15074 31222782

[14] Kim MJ Kim EK Kim BM Kwak JY Lee EJ Park CS. Thyroglobulin measurement in fine-needle aspirate washouts: the criteria for neck node dissection for patients with thyroid cancer. Clin Endocrinol (Oxf) (2009) 70(1):145–51. doi: 10.1111/j.1365-2265.2008.03297.x 18466347

[15] Whiting PF Rutjes AW Westwood ME Mallett S Deeks JJ Reitsma JB. QUADAS-2: A revised tool for the quality assessment of diagnostic accuracy studies. Ann Intern Med (2011) 155(8):529–36. doi: 10.7326/0003-4819-155-8-201110180-00009 22007046

[16] Higgins JP Altman DG Gøtzsche PC Jüni P Moher D Oxman AD. The cochrane collaboration's tool for assessing risk of bias in randomised trials. Bmj (2011) 343:d5928. doi: 10.1136/bmj.d5928 22008217 PMC3196245

[17] Salmaslioglu A Erbil Y Citlak G Ersoz F Sari S Olmez A. Diagnostic value of thyroglobulin measurement in fine-needle aspiration biopsy for detecting metastatic lymph nodes in patients with papillary thyroid carcinoma. Langenbecks Arch Surg (2011) 396(1):77–81. doi: 10.1007/s00423-010-0723-1 21052711

[18] de Crea C Raffaelli M Maccora D Carrozza C Canu G Fadda G. Calcitonin measurement in fine-needle aspirate washouts vs. cytologic examination for diagnosis of primary or metastatic medullary thyroid carcinoma. Acta Otorhinolaryngol Ital (2014) 34(6):399–405. 25762832 PMC4346997

[19] Byun BH Jeong U-G Hong SP Min JJ Chong A Song H-C. Prediction of central lymph node metastasis from papillary thyroid microcarcinoma by f-18-fluorodeoxyglucose PET/CT and ultrasonography. Ann Nucl Med (2012) 26(6):471–7. doi: 10.1007/s12149-012-0594-3 22467230

[20] de Crea C Raffaelli M Maccora D Carrozza C Canu G Fadda G. Calcitonin measurement in fine-needle aspirate washouts vs. cytologic examination for diagnosis of primary or metastatic medullary thyroid carcinoma. Acta Otorhinolaryngol Ital (2014) 34(6):399–405. 25762832 PMC4346997

[21] Samanci C Onal Y Sager S Asa S Ustabasioglu FE Alis D. Diagnostic capabilities of MRI versus f-18 FDG PET-CT in postoperative patients with thyroglobulin positive, I-131-negative local recurrent or metastatic thyroid cancer. Curr Med Imaging (2019) 15(10):956–64. doi: 10.2174/1573405614666180718124739 32008523

[22] Lee JH Lee HC Yi HW Kim BK Bae SY Lee SK. Influence of thyroid gland status on the thyroglobulin cutoff level in washout fluid from cervical lymph nodes of patients with recurrent/metastatic papillary thyroid cancer. Head Neck-Journal Sci Specialties Head Neck (2016) 38:E1705–12. doi: 10.1002/hed.24305 26614648

[23] David L Cunnane ME Zurakowski D Acar G Ecevit C Mace A. Papillary thyroid carcinoma nodal surgery directed by a preoperative radiographic map utilizing CT scan and ultrasound in all primary and reoperative patients. Head Neck-Journal Sci Specialties Head Neck (2014) 36(2):191–202. 10.1002/hed.23277 23554058

[24] Baldini E Sorrent S Di Gioia C De Vito C Antonelli A Gnessi L. Cervical lymph node metastases from thyroid cancer: Does thyroglobulin and calcitonin measurement in fine needle aspirates improve the diagnostic value of cytology? BMC Clin Pathol (2013) 13:7–7. doi: 10.1186/1472-6890-13-7 23421519 PMC3583740

[25] Eun NL Son EJ Kim JA Gweon HM Kang JH Youk JH. Comparison of the diagnostic performances of ultrasonography, CT and fine needle aspiration cytology for the prediction of lymph node metastasis in patients with lymph node dissection of papillary thyroid carcinoma: A retrospective cohort study. Int J Surg (2018) 51:145–50. doi: 10.1016/j.ijsu.2017.12.036 29337176

[26] Farzana A Naito K Horiguchi J Fukuda H Tachikake T Ito K. Accuracy of sonographic elastography in the differential diagnosis of enlarged cervical lymph nodes: Comparison with conventional b-mode sonography. Am J Roentgenology (2008) 191(2):604–10. 10.2214/AJR.07.3401 18647939

[27] Jeong HS Baek CH Son YI Choi JY Kim HJ Ko YH. Integrated 18F-FDG PET/CT for the initial evaluation of cervical node level of patients with papillary thyroid carcinoma: Comparison with ultrasound and contrast-enhanced CT. Clin Endocrinol (Oxf) (2006) 65(3):402–7. doi: 10.1111/j.1365-2265.2006.02612.x 16918964

[28] Hwang H Perez D Orloff L. Comparison of positron emission Tomography/Computed tomography imaging and ultrasound in staging and surveillance of head and neck and thyroid cancer. Laryngoscope (2009) 119(10):1958–65. doi: 10.1002/lary.20594 19688854

[29] Mihailovic J Prvulovic M Ivkovic M Markoski B Martinov D. MRI Versus I-131 whole-body scintigraphy for the detection of lymph node recurrences in differentiated thyroid carcinoma. Am J Roentgenology (2010) 195(5):1197–203. doi: 10.2214/AJR.09.4172 20966328

[30] Jeon SJ Kim E Park JS Son KY Baek J Kim YS. Diagnostic benefit of thyroglobulin measurement in fine-needle aspiration for diagnosing metastatic cervical lymph nodes from papillary thyroid cancer: Correlations with US features. Korean J Radiol (2009) 10(2):106–11. doi: 10.3348/kjr.2009.10.2.106 PMC2651448 19270855

[31] Choi YJ Yun JS Kook SH Jung EC Park YL. Clinical and imaging assessment of cervical lymph node metastasis in papillary thyroid carcinomas. World J Surg (2010) 34(7):1494–9. doi: 10.1007/s00268-010-0541-1 20372903

[32] Jung JH Kim CY Son SH Kim DH Jeong SY Lee SW. Preoperative prediction of cervical lymph node metastasis using primary tumor SUVmax on 18F-FDG PET/CT in patients with papillary thyroid carcinoma. PloS One (2015) 10(12):e0144152. doi: 10.1371/journal.pone.0144152 26636824 PMC4670197

[33] Zhan J Diao X-H Chen YC Wang W-P Ding H. Homogeneity parameter in contrast-enhanced ultrasound imaging improves the classification of abnormal cervical lymph node after thyroidectomy in patients with papillary thyroid carcinoma. BioMed Res Int (2019) 26(2019):9296010. doi: 10.1155/2019/9296010 PMC6899314 31886269

[34] Zhan J Diao X-H Chen YC Wang W-P Ding H. Homogeneity parameter in contrast-enhanced ultrasound imaging improves the classification of abnormal cervical lymph node after thyroidectomy in patients with papillary thyroid carcinoma. BioMed Res Int (2019) 2019. doi: 10.1155/2019/9296010 PMC6899314 31886269

[35] Lee DW Ji YB Sung ES Park JS Lee YJ Park DW. Roles of ultrasonography and computed tomography in the surgical management of cervical lymph node metastases in papillary thyroid carcinoma. Ejso (2013) 39(2):191–6. doi: 10.1016/j.ejso.2012.07.119 22863305

[36] Khadra H Mohamed H Al-Qurayshi Z Sholl A M Emad Kandil E. Superior detection of metastatic cystic lymphadenopathy in patients with papillary thyroid cancer by utilization of thyroglobulin washout. Head Neck-Journal Sci Specialties Head Neck (2019) 41(1):225–9. doi: 10.1002/hed.25488 30536535

[37] Li J Zhang K Liu X Hao F Liu Z Wang Z. Cervical lymph node thyroglobulin measurement in washout of fine-needle aspirates for diagnosis of papillary thyroid cancer metastases. Br J BioMed Sci (2016) 73(2):79–83. doi: 10.1080/09674845.2016.1173334 27181176

[38] Shi JH Xu YY Pan QZ Sui GQ Zhou JP Wang H. The value of combined application of ultrasound-guided fine needle aspiration cytology and thyroglobulin measurement for the diagnosis of cervical lymph node metastases from thyroid cancer. Pak J Med Sci (2015) 31(5):1152–5. doi: 10.12669/pjms.315.6726 PMC4641273 26649004

[39] Yang SY Shin JH Hahn SY Lim Y Hwang SY Kim TH. Comparison of ultrasonography and CT for preoperative nodal assessment of patients with papillary thyroid cancer: diagnostic performance according to primary tumor size. Acta Radiol (2020) 61(1):21–7. doi: 10.1177/0284185119847677 31084186

[40] Sohn YM Kim MJ Kim EK Kwak JY. Diagnostic performance of thyroglobulin value in indeterminate range in fine needle aspiration washout fluid from lymph nodes of thyroid cancer. Yonsei Med J (2012) 53(1):126–31. doi: 10.3349/ymj.2012.53.1.126 PMC3250316 22187242

[41] Suh YJ Son EJ Moon HJ Kim EK Han K Kwak JY. Utility of thyroglobulin measurements in fine-needle aspirates of space occupying lesions in the thyroid bed after thyroid cancer operations. Thyroid (2013) 23(3):280–8. doi: 10.1089/thy.2011.0303 22950788

[42] Liu T Ge X Yu J Guo Y Wang Y Wang W. Comparison of the application of b-mode and strain elastography ultrasound in the estimation of lymph node metastasis of papillary thyroid carcinoma based on a radiomics approach. Int J Comput Assisted Radiol Surg (2018) 13(10):1617–27. doi: 10.1007/s11548-018-1796-5 29931410

[43] Wang Y Nie F Wang G Liu T Dong T Sun Y. Value of combining clinical factors, conventional ultrasound, and contrast-enhanced ultrasound features in preoperative prediction of central lymph node metastases of different sized papillary thyroid carcinomas. Cancer Manag Res (2021) 13:3403–15. doi: 10.2147/CMAR.S299157 PMC8064616 33907464

[44] Liu Y Li S Yan C He C Yun M Liu M. Value of dual-phase, contrast-enhanced CT combined with ultrasound for the diagnosis of metastasis to central lymph nodes in patients with papillary thyroid cancer. Clin Imaging (2021) 75:5–11. doi: 10.1016/j.clinimag.2021.01.008 33486150

[45] Wei Y Yu M Niu Y Hao Y Di J Zhao Z. Combination of Lymphatic and Intravenous Contrast-Enhanced Ultrasound for Evaluation of Cervical Lymph Node Metastasis from Papillary Thyroid Carcinoma: A Preliminary Study. Ultrasound Med Biol (2021) 47(2):252–60. doi: 10.1016/j.ultrasmedbio.2020.10.003 33158634

[46] Hong YR Luo ZY Mo GQ Wang P Ye Q Huang PT. Role of Contrast-Enhanced Ultrasound in the Pre-operative Diagnosis of Cervical Lymph Node Metastasis in Patients with Papillary Thyroid Carcinoma. Ultrasound Med Biol (2017) 43(11):2567–75. doi: 10.1016/j.ultrasmedbio.2017.07.010 28807450

[47] Liu Z Xun X Wang Y Mei L He L Zeng W. MRI And ultrasonography detection of cervical lymph node metastases in differentiated thyroid carcinoma before reoperation. Am J Trans Res (2014) 6(2):147–54. PMC3902225 24489994

[48] Zhao H Wang Y Wang MJ Zhang ZH Wang HR Zhang B. Influence of presence/absence of thyroid gland on the cutoff value for thyroglobulin in lymph-node aspiration to detect metastatic papillary thyroid carcinoma. BMC Cancer (2017) 17(1):296. doi: 10.1186/s12885-017-3296-3 28454525 PMC5410021

[49] Zhao H Li H. Meta-analysis of ultrasound for cervical lymph nodes in papillary thyroid cancer: Diagnosis of central and lateral compartment nodal metastases. Eur J Radiol (2019) 112:14–21. doi: 10.1016/j.ejrad.2019.01.006 30777203

[50] Morita S Mizoguchi K Suzuki M Iizuka K. The accuracy of (18)[F]-fluoro-2-deoxy-D-glucose-positron emission tomography/computed tomography, ultrasonography, and enhanced computed tomography alone in the preoperative diagnosis of cervical lymph node metastasis in patients with papillary thyroid carcinoma. World J Surg (2010) 34(11):2564–9. doi: 10.1007/s00268-010-0733-8 20645089

[51] Sheng L Shi J Han B Lv B Li L Chen B. Predicting factors for central or lateral lymph node metastasis in conventional papillary thyroid microcarcinoma. Am J Surg (2020) 220(2):334–40. doi: 10.1016/j.amjsurg.2019.11.032 31818425

[52] Gu JH Zhao YN Xie RL Xu WJ You DL Zhao ZF. Analysis of risk factors for cervical lymph node metastasis of papillary thyroid microcarcinoma: A study of 268 patients. BMC Endocr Disord (2019) 19(1):124. doi: 10.1186/s12902-019-0450-8 31729977 PMC6858775

[53] Medas F Canu GL Cappellacci F Boi F Lai ML Erdas E. Predictive factors of lymph node metastasis in patients with papillary microcarcinoma of the thyroid: Retrospective analysis on 293 cases. Front Endocrinol (Lausanne) (2020) 11:551. doi: 10.3389/fendo.2020.00551 32982963 PMC7477034

[54] Loevner L Kaplan S Cunnane M-E Moonis G. Cross-sectional imaging of the thyroid gland. Neuroimaging Clinics North America (2008) 18(3):445–61. doi: 10.1016/j.nic.2008.05.001 18656027

[55] Yu XM Yang XY Qiu JC Zhu YH Zhou T Lin J. Analysis of current situation of CT and MRI examination service price items. China Med Equip (2021) 18(04):168–171.

[56] Paul AB Oklu R Saini S Prabhakar AM. How much is that head CT? price transparency and variability in radiology. J Coll Radiol (2015) 12(5):453–7. doi: 10.1016/j.jacr.2014.12.016 25841864

[57] Sinaiko AD Rosenthal MB. Increased price transparency in health care–challenges and potential effects. N Engl J Med (2011) 364(10):891–4. doi: 10.1056/NEJMp1100041 21388306

